# Investigating the Microstructure and Mechanical Properties of Aluminum-Matrix Reinforced-Graphene Nanosheet Composites Fabricated by Mechanical Milling and Equal-Channel Angular Pressing

**DOI:** 10.3390/nano9081070

**Published:** 2019-07-25

**Authors:** Mahdi Hasanzadeh Azar, Bahareh Sadri, Alireza Nemati, Shayan Angizi, Mohammad Hossein Shaeri, Peter Minárik, Jozef Veselý, Faramarz Djavanroodi

**Affiliations:** 1 Department of Materials Science and Engineering, Imam Khomeini International University (IKIU), Qazvin 3414916818, Iran; 2 Department of Chemistry and Chemical Biology, McMaster University, Hamilton, ON L8S 4M1, Canada; 3 Department of Physics of Materials, Faculty of Mathematics and Physics, Charles University, Ke Karlovu 5, Praha 2 121 16, Czech Republic; 4 Mechanical Engineering Department, Prince Mohammad Bin Fahd University, Al Khobar 31952, Saudi Arabia; 5 Department of Mechanical Engineering, Imperial College London, London SW7, UK

**Keywords:** aluminum matrix, graphene nanosheets, equal-channel angular pressing (ECAP), ball milling, mechanical properties, microstructure

## Abstract

Layered-graphene reinforced-metal matrix nanocomposites with excellent mechanical properties and low density are a new class of advanced materials for a broad range of applications. A facile three-step approach based on ultra-sonication for dispersion of graphene nanosheets (GNSs), ball milling for Al-powder mixing with different weight percentages of GNSs, and equal-channel angular pressing for powders’ consolidation at 200 °C was applied for nanocomposite fabrication. The Raman analysis revealed that the GNSs in the sample with 0.25 wt.% GNSs were exfoliated by the creation of some defects and disordering. X-ray diffraction and microstructural analysis confirmed that the interaction of the GNSs and the matrix was almost mechanical, interfacial bonding. The density test demonstrated that all samples except the 1 wt.% GNSs were fully densified due to the formation of microvoids, which were observed in the scanning electron microscope analysis. Investigation of the mechanical properties showed that by using Al powders with commercial purity, the 0.25 wt.% GNS sample possessed the maximum hardness, ultimate shear strength, and uniform normal displacement in comparison with the other samples. The highest mechanical properties were observed in the 0.25 wt.% GNSs composite, resulting from the embedding of exfoliated GNSs between Al powders, excellent mechanical bonding, and grain refinement. In contrast, agglomerated GNSs and the existence of microvoids caused deterioration of the mechanical properties in the 1 wt.% GNSs sample.

## 1. Introduction

Recently, graphene nanosheets (GNSs), with 2D honeycomb structure, and their derivatives have gained dramatic attention due to their unique properties such as a high mechanical strength [1], chemical stability, excellent electrical and thermal conductivity [2], as well as biocompatibility [3,4,5]. It is proved that the quality of GNSs and their applications are completely related to their fabrication process [6]. A broad range of fabrication methods from top-down approaches, such as mechanical exfoliation of graphite [7], to bottom-up approaches, such as chemical vapor deposition (CVD) [6,8,9] have been utilized to produce high-quality graphene with large area and atomic thickness [10]. As the product’s functionality is completely associated with the fabrication method, careful selection of the proper approach for each application is required [3].

One of the first breakthroughs in graphene application was reinforcing the nanocomposites and, consequently, improving the different properties such as high specific mechanical strength or the thermal properties [11,12,13]. However, other aspects of graphene have made it suitable for many other applications. For example, Pala et al. reported that the PLA/CNC/rGO (poly lactic acid/cellulose nanocrystal/reduced graphene oxide) nanocomposite is a promising material for the manufacturing of scaffolds considering the biomedical properties of graphene [14]. Also, Huang et al. demonstrated that dispersing exfoliated graphene oxide nanosheets (GONs) in the PLA-film matrix lead to improvement of the film’s barrier properties and also a permeability decrement of O_2_ and CO_2_ gases [15,16]. On the other hand, the investigation on reinforced Al_2_O_3_ ceramic matrices by graphene platelets showed that graphene not only causes a dramatic increase in toughness, but also provides a higher fracture strength [17]. Moreover, Fan et al. reported that due to the presence of GNSs in the Al_2_O_3_ matrix, the conductivity of the nanocomposites could be significantly increased [18]. Also, it was found that employing a single layer of graphene instead of multiple layers leads to a considerable improvement in mechanical properties of the silicon carbide matrix in M. Barfmal’s study [19].

Recently, the low-weight metal-matrix nanocomposites have attracted much interest, because they provide a low-density structure, excellent electrical conductivity, and high mechanical properties simultaneously [20,21,22]. Among them, the aluminum-matrix nanocomposites have been widely investigated owing to advantageous properties such as a high strength/weight numerical ratio, flexibility, adaptable corrosion resistance, conductivity, and availability [23,24]. Furthermore, their applicability can be proven by their vast usage in commercial and military applications such as in helicopter instruments, the cargo bay of space shuttles, flywheels, and the retainer rings used in high-speed motors [25]. Many of these features can be achieved when the reinforcements are completely dispersed in the matrices. Therefore, complete dispersion and uniform distribution of GNSs in the Al matrix as a result of their plate shapes and surface functionality [26] make them appealing for many applications, while other carbon-based nanostructures such as CNTs (carbon nanotubes) cannot be distributed perfectly due to their cylindrical shape [27]. In 2015, Rashad et al. proposed a fabrication technique of Al/GNP nanocomposites using powder metallurgy approaches and subsequent hot extrusion processes that led to a 13.5% increase in the ultimate tensile strength and a 50% enhancement in the failure strain in comparison with the pure matrix [26]. In another study, researchers utilized high-energy ball milling and vacuum hot pressing for the fabrication of Al-based nanocomposites reinforced by GNSs. Interestingly, Vickers hardness increased up to 81 in the 1 wt.% GNSs sample, and the researchers also observed that yield strength of the sample with 0.25 wt.% graphene increased by 38.27% in comparison with that of the pure Al [28]. In 2017, Saboori et al. fabricated an Al–graphene nanocomposite by using powder metallurgy and subsequent hot rolling techniques. The results showed a remarkable drop in density, while Vickers hardness was comparable to samples produced by pressing and sintering. Also, thermal conductivity decreased slightly in comparison with the press-sintered product [29].

Despite intensive research in this field, finding a reliable method for fabrication of nanocomposites is still a challenge. Among newly developed approaches, severe plastic deformation (SPD) is a promising technique for the introduction of a high number of high-angle grain boundaries by imposing a large plastic strain [30]. Today, several SPD techniques including equal-channel angular pressing (ECAP) [31], torsion extrusion (TE) [32], accumulative roll-bonding (ARB) [33], multi-directional forging (MDF) [34], and high-pressure torsion (HPT) [35] are available. To the best of the authors’ knowledge, there are only a few reports on the fabrication of Al-GNS composites using these SPD approaches. Zhao et al. attained 122 HV hardness in the sample with 0.5 wt.% graphene that was embedded in the Al matrix by the HPT process. Surprisingly, the density of this sample was above 98% [36]. In the current study, new route, including ultra-sonication and ball milling, was explored for dispersing GNSs in the Al matrix. Subsequently, in comparison with the above-mentioned processes, ECAP was utilized for the consolidation of samples. This method is one of the most facile and effective SPD methods for fabricating nanostructured-grain bulk materials [37,38,39,40]. Excellent features such as an ultrafine-grain (UFG) and nanocrystalline (NC) structure are produced by the SPD methods, which usually result in significant improvement of strength and toughness [41]. In addition, for the first time, we produced the bulk of the Al-GNS nanocomposites by just using the shear stress in the intersecting channels with only one pass at a temperature of 200 °C. The products were investigated by different characterization techniques including transmission electron microscopy (TEM), X-ray diffraction (XRD), scanning electron microscopy (SEM), Raman spectroscopy, as well as microhardness and shear punch tests. The results show that the Al-0.25 wt.% GNS composite produced by the ECAP process showed desirable mechanical properties that are comparable with published results for Al-GNS composites produced by other processes [26,27,28,36,42].

## 2. Materials and Methods 

### 2.1. Materials

**Figure 1:**
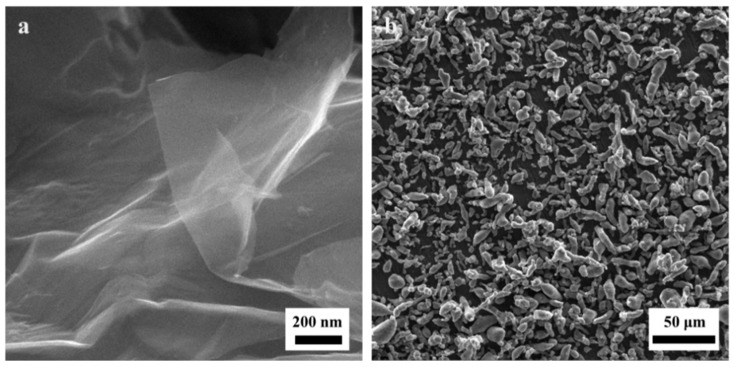
SEM images of (**a**) pure graphene nanosheets and (**b**) aluminum powders.

The GNSs, also known as few-layer graphenes, with a specific surface area of 350 g/m^2^ and a lateral size of no more than 5 micrometers were supplied by AVANSA Technology & Services, Uttar Pradesh, India. Aluminum powders with commercial purity (purity 99%, lateral size less than 45 micrometers) were bought from Alfa Aesar, Karlsruhe, Germany. SEM images of GNSs and Al powders are shown in Figure 1a,b. 

### 2.2. Experimental Procedure

#### 2.2.1. Powder Preparation 

As-bought GNSs were dispersed in (100 mL) ethanol using an ultrasonic bath (35 kHz) for 1 h and then were mixed with aluminum powders in order to fabricate 0.1, 0.25, 0.5, and 1 weight percent of Al-GNS nanocomposites. The mixture was put in a high-energy ball milling for 4 h at 360 RPM with the ball to powder ratio of 10:1. Moreover, Ar gas was introduced to the ball milling to avoid any oxidation and contamination. Finally, the ball-milled mixture was dried in a vacuum oven for 19 h at 90 °C to prepare the Al-GNS powders.

#### 2.2.2. ECAP Process

**Figure 2:**
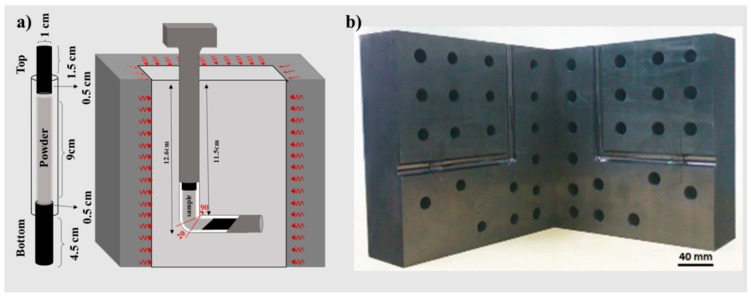
(**a**) Schematic of the consolidation process and (**b**) the equal-channel angular pressing (ECAP) die used in the current research.

In order to consolidate the as-prepared mixture, the mixed powders were pressed using an ECAP die according to Figure 2a. As depicted in Figure 2b, the steel die possess horizontal and vertical channels, which intersect at an angle of φ = 90° with a corner curvature of ψ = 20°. In addition, the height and diameter of the die channels were 126 mm and 12.3 mm, respectively. For reduction of the produced heat and the friction between the sample and mold, copper tube casings were employed to avoid the creation of dead parts in the samples as well as to keep the powder particles together. Furthermore, two short bars were inserted in the bottom and top of the copper tube, and the tube was filled with mixed powders. Before compacting the powders, the sample and the die were heated at a heating rate of 0.83 °C/min for 2 h to reach 200 °C. Then, the compaction was performed by pressing through the ECAP die with a strain rate of 1.5 mm·s^−1^. Finally, the samples created by ECAP were cooled naturally.

### 2.3. Characterization

After fabrication of the samples by ECAP method, Raman spectroscopy was applied for identifying the level of GNS disordering. The changes in thickness of the GNS layers for both powder and bulk samples were measured by an Nd:Yag laser in at least three areas from each sample (laser wavelength: 532 nm, power of laser: 5 mW, Co: Teksan type: Takram P50C0R10). Calculation of the ID/IG parameter was based on the ratio of the maximum intensity of the D peak (ID) to the maximum intensity of the G peak (IG). X-ray diffraction analysis was carried out to characterize the phase composition (PHILIPS Co type: PW1730, I = 30 mA, V = 40 kV). Light optical microscopy (LOM) (BX60MF5, Olympus Optical Co., Tokyo, Japan) was utilized for initial observation of the microstructure. To characterize the microporosities and microcracks and to investigate the agglomeration and distribution of GNSs, as well as the creation of refined grains, scanning electron microscopy (SEM) with energy dispersive spectrometer (MIRA II LMU, TeScan Co.) and transmission electron microscopy (Jeol 2200FS) were used. For TEM characterization, the disc-shaped samples were mechanically thinned and afterwards ion-polished using a Leica EM RES102 system. The density of all consolidated samples was calculated three times from each sample using the Archimedes’ principle by weighting the nanocomposite samples in the air as well as after dipping in 100 °C distilled water for 2 h.

Vickers microhardness test (HVS-1000A instrument, Laizhou Lyric Testing Equipment Co.) was performed through the composite cross-section in order to analyze homogeneity of mechanical properties. At least five measurements were performed for each position with an applied weight of 500 g for 15 s. A shear punch test was conducted three times for each sample for calculating shear strength and normalized displacement under shear force with a strain rate of 10^−3^ s^−1^ (Zwick/Roell Company, type: BCT-EXMACRO.001). The details of the shear punch test and the procedure for the shear strength and normalized displacement measurements were presented in previous papers [43,44,45]. 

## 3. Results

### 3.1. X-Ray Diffraction

**Figure 3:**
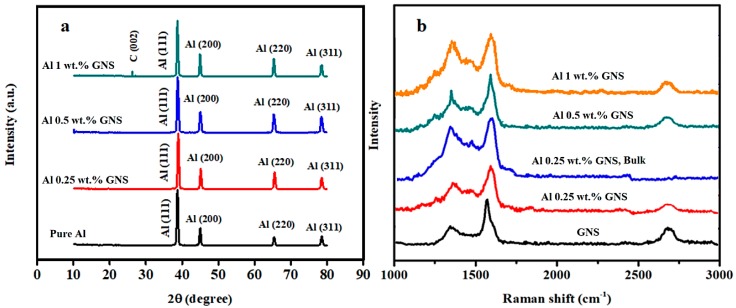
(**a**) X-ray diffraction patterns of aluminum and consolidated Al/graphene nanosheet (GNS) nanocomposites; (**b**) Raman spectra of pure GNSs, the ball milled Al-GNS powders with different weight percent, and consolidated 0.25 wt.% Al-GNS nanocomposite (bulk).

X-ray diffraction patterns of pure aluminum and its nanocomposites with different weight percent of GNSs are shown in Figure 3a. As can be seen, aluminum peaks of (111), (200), (220). and (311) planes are present at 38.68°, 44.98°, 65.37°, and 78.52° angles, respectively. As expected, no peaks related to GNSs were observed in any samples except the 1 wt.% GNSs, in which a small peak corresponding to graphene (C) occurred. The occurrence of small or negligible graphene peaks is a consequence of the low content of GNSs in the matrix and their nanoscale dimensionality [46]. It should be noted that no Al_2_O_3_ and/or Al_4_C_3_ peaks were observed, which are typical for comparable materials. It has been reported that graphene could react with Al at high temperatures during fabrication of a composite to form an Al_4_C_3_ phase. Moreover, the high temperature could cause disordering of graphene particles, leading to the deterioration of their beneficial properties [47]. In addition, Al_2_O_3_ and Al_4_C_3_ particles are nucleation sites for microcracks formation and cause a decrease in the strength of the Al-GNS nanocomposites [28]. Since the processing temperature of ECAP in the current study was relatively low, the formation of the Al_4_C_3_ phase was not expected. On the other hand, a low concentration of Al_2_O_3_ cannot be disregarded. A small-volume fraction of oxides is usually formed during ball-milling, ECAP, and manipulation with powder material. As shown later, Al_2_O_3_ is present in all consolidated samples along the former particles’ boundaries. Nevertheless, its amount is below XRD resolution.

### 3.2. Raman Spectroscopy

Raman spectroscopy was carried out to characterize the level of disordering of the GNSs and the changing number of their layers in samples containing different weight percentages of GNSs prior to and after ECAP. According to a previous report [48], the optimized time for ball-milling of Al-GNS nanocomposites is 4 h. This time not only provides a reasonably homogeneous distribution of GNSs, but also has a positive effect on resisting an increase of the GNSs’ amorphousity. As can be observed in Figure 3b, the Raman spectrum of pure GNSs exhibits a small D band at 1345 cm^−1^, strong G band at 1571 cm^−1^, and a second order 2D band at 2680 cm^−1^. In addition, the presence of the D band in the GNSs showed that some defects and impurities existed in the primary GNSs [49]. According to Table 1, a small blue shift of the G peak in all ball-milled samples clearly revealed the disruption of the agglomeration of GNSs as well as a decrease in the number of their layers. Moreover, the increase in the GNSs weight percent did not cause a change in the peak position owing to the same duration of ball-milling in all samples. On the other hand, a small blue shift observed in the G peak of the 0.25 wt.% GNS bulk sample, when compared to its powder counterpart, indicates that graphene nanosheet layers were probably exfoliated. As previously discussed by Zare et al. [27], dislocations in aluminum could accumulate behind the carbon reinforcements during ECAP. Therefore, it is reasonable to expect that the shear stress imposed by accumulated dislocations can outweigh the Van der Walls interaction between the layers of some GNSs and consequently lead to a decrease in the number of graphene layers. This process would predominantly occur in GNS oriented parallel to shear stress direction during ECAP. 

The intensity ratio of D (ID) and G (IG) peaks (ID/IG), which is essential for the characterization of GNS disorder and other attributes [50], is given for all samples in Table 1. First, an increase in this parameter with increasing weight percent of the GNSs (powder samples) can be attributed to the higher disordering of the GNSs due to a higher number of collisions between balls and GNSs. Therefore, a lower value of this parameter for the 0.25 wt.% GNS sample in comparison with those of the 0.5 and 1 wt.% GNS samples reveals that the crystal structure of the GNSs in the 0.25 wt.% GNS sample is more preserved after milling. Also, an increase in this parameter in the consolidated 0.25 wt.% GNS sample resulted from the stresses applied during processing. However, the increment of this parameter for the 0.25 wt.% GNS bulk sample was lower in comparison with that of the Al/graphene nanosheet composites fabricated by different techniques at higher temperatures [47,51]. In addition, the 1 wt.% GNS powder had the highest amount of ID/IG, and it is evident that the bulk sample of 1 wt.% GNS had a higher amount of this parameter. Therefore, it is probable that some GNSs were oxidized by mechanochemical reactions [52,53] during manipulation with powders in the 1 wt.% GNS bulk sample, which will be discussed in the SEM section [54,55].

### 3.3. Microstructure Characterization

#### 3.3.1. Light Optical Microscopy

**Figure 4:**
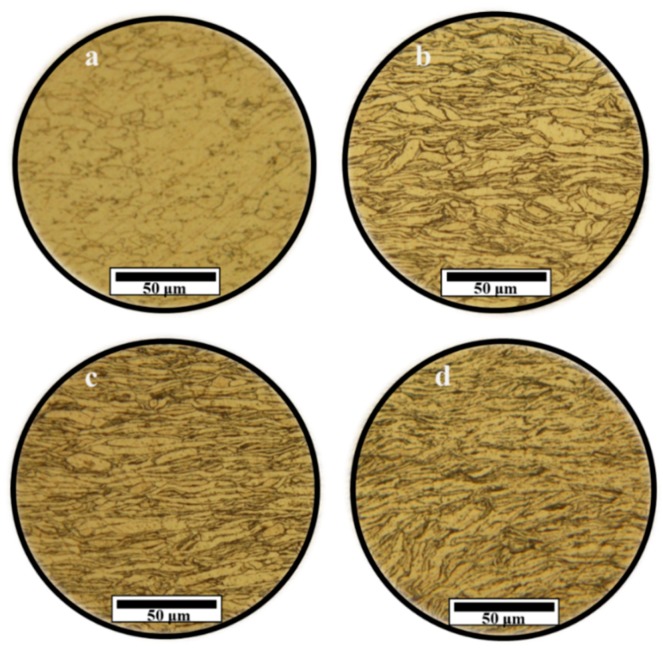
LOM images of ECAPed specimens after etching: (**a**) Aluminum; (**b**) Al-0.25 wt.% GNS; (**c**) Al-0.5 wt.% GNS; and (**d**) Al-1 wt.% GNS.

LOM was performed after etching the specimens’ surface. Figure 4a–d shows the etched microstructure of the investigated consolidated samples. Two crucial pieces of information are noteworthy. The first is that the former aluminum flake-like particles created during ball-milling were stretched along the theoretical shear plane of ECAP during the processing. The formation of shear bands oriented parallel to the theoretical shear plane of ECAP was already observed previously in different materials processed similarly. The other point is that the average particle size decreased with increasing GNS content. GNSs act as an effective barrier and help to prevent cold-welding of Al-powder particles during ball-milling [48]. The size of the Al powders decreased at the initial stage of ball milling, and with the passage of time, they were cold-welded and their size increased. However, GNSs can operate as a barrier against cold-welding and, consequently, the increasing GNSs content decreases the powder size. 

#### 3.3.2. Scanning Electron Microscopy 

**Figure 5:**
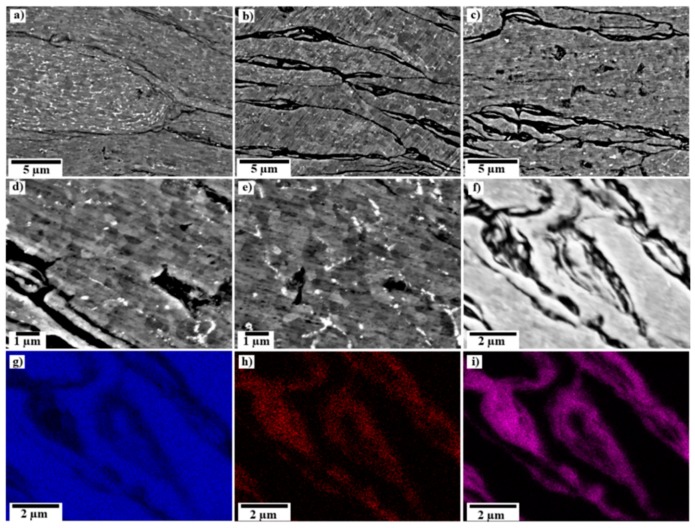
SEM images acquired from the center of consolidated (**a**) Al; (**b**) 0.25 wt.% GNSs; and (**c**) 1 wt.% GNS samples. Detail of the microstructure of the 1 wt.% GNS sample; (**d**) central part; and (**e**) outer perimeter. Elemental distribution maps of (**g**) Al; (**h**) C; and (**i**) O elements in the 1 wt.% GNS sample from the area shown in (**f**).

Figure 5a–c shows a microstructure of pure aluminum, 0.25 wt.%, and 1 wt.% GNS samples using SEM. The composition as measured by Energy-dispersive X-ray spectroscopy (EDS) in SEM revealed that contaminations of Si (~0.5 wt.%) and Fe (~0.4 wt.%) were present in the pure aluminum, which resulted in the formation of typical (Al-)Fe-Si second phase particles. These results are consistent with the declared grade of purity of the initial aluminum powders. The distribution of contamination is not homogenous concerning individual powder particles, as seen in Figure 5a. Most of the grains contain only a low content of secondary phases, but the small-volume fractions contain a cellular substructure of secondary phases resulting from the dendritic solidification. As can be seen, the thickness of the boundary of the powders was very low in the consolidated pure aluminum; while by adding GNSs to the pure Al incrementally, the thickness of the boundaries increased, and the boundary thickness reached the highest amount in the sample containing 1 wt.% GNSs. 

Additionally, a difference in the grain size was observed between the center and outer perimeter of all consolidated samples. As shown in Figure 5d,e, the average grain size increased from ~300 nm to ~500 nm in the 1 wt.% GNS sample. This variation of grain-size distribution through all samples resulted in variation of the microhardness, as shown later. 

Local analysis of the elemental distribution of the 1 wt.% GNS sample in Figure 5f revealed increased oxygen and carbon along the former particle boundaries. A corresponding EDS maps of the 1 wt.% GNS sample is shown in Figure 5g–i. The higher oxygen content can be a result of the formation of Al_2_O_3_, as well as the occurrence of oxidization of some GNSs during manipulation with the powder, which is proven with the high values of ID/IG for this sample [54,55]; nevertheless, as shown by XRD measurements, a small peak corresponding to graphene and the absence of Al_2_O_3_ peaks demonstrated that the volume fraction was low. In addition, it is reasonable that GNSs would be present in the powder’s boundaries due to the low diffusion of carbon as well as the low solubility of carbon in Al at the processing temperature (200 °C). However, the thick boundaries, higher content of carbon, and creation of microvoids in the Al powders can be explained by the increased possibility of the agglomeration of GNSs along the former powder boundaries, which will be shown in the TEM section. It is noteworthy to mention that when the percentage of GNSs reached 1 wt.%, more parts of the GNS particles attached to the powder surface during milling and did not distribute to the aluminum matrix during milling and subsequent consolidation. Therefore, microvoids were created in the matrix and at powder boundaries due to the agglomeration of GNSs along the former powder boundaries. 

#### 3.3.3. Transmission Electron Microscopy

**Figure 6:**
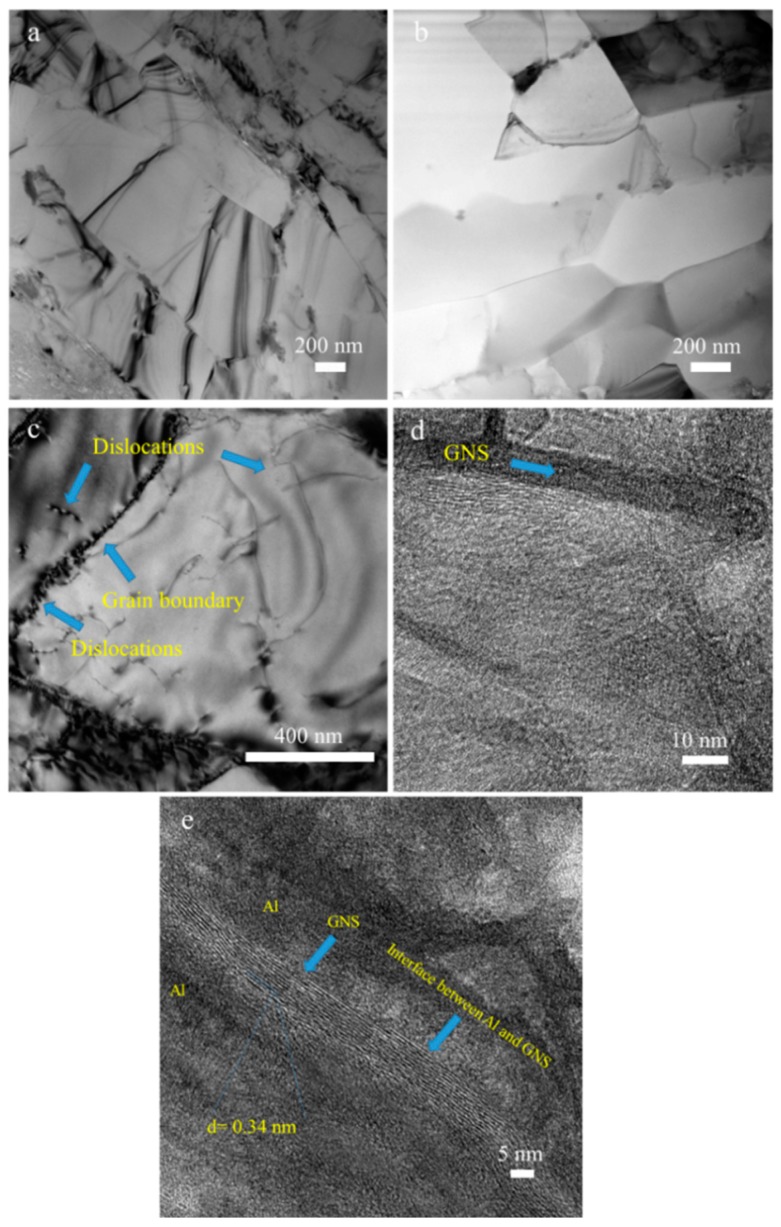
Transmission electron microscopic images of (**a**) Al-1 wt.% GNS; (**b**,**c**) Al-0.25 wt.% GNS. (**d**,**e**) High-resolution transmission electron microscopic image of Al-0.25 wt.% GNSs.

TEM was used to observe and identify GNSs inside the Al matrix. The TEM micrographs of the samples with 0.25 wt.% and 1 wt.% of the GNSs are shown in Figure 6a,b, respectively. Formation of the ultrafine-grained structure in the consolidated samples observed by SEM was also proven by TEM. As mentioned, grain refinement through severe plastic deformation is one of the most important features occurring during ECAP [56]. In Figure 6c, one of the grain boundaries and dislocations in the 0.25 wt.% GNS composite has been marked by an arrow. Interaction between dislocations; dislocation pile-ups behind grain boundaries or Al-GNS interfaces; and formation of cellular walls or sub-grains are the main reasons for dynamic and static recrystallization and consequent improvement of mechanical properties. 

Figure 6d,e shows high-resolution images of the sample with the 0.25 wt.% graphene nanosheet in higher magnification. The dark part, with a thickness of less than 3 nm, which was located in the Al matrix, was identified as a GNS. For verification of this claim, the interplanar distance of the GNS was measured with a result of 0.34 nm, which is precisely equal to the distance between the graphene plates [57], see Figure 6e. In addition, the defect-free mechanical bond between the exfoliated GNS and the Al matrix was observed. 

**Figure 7:**
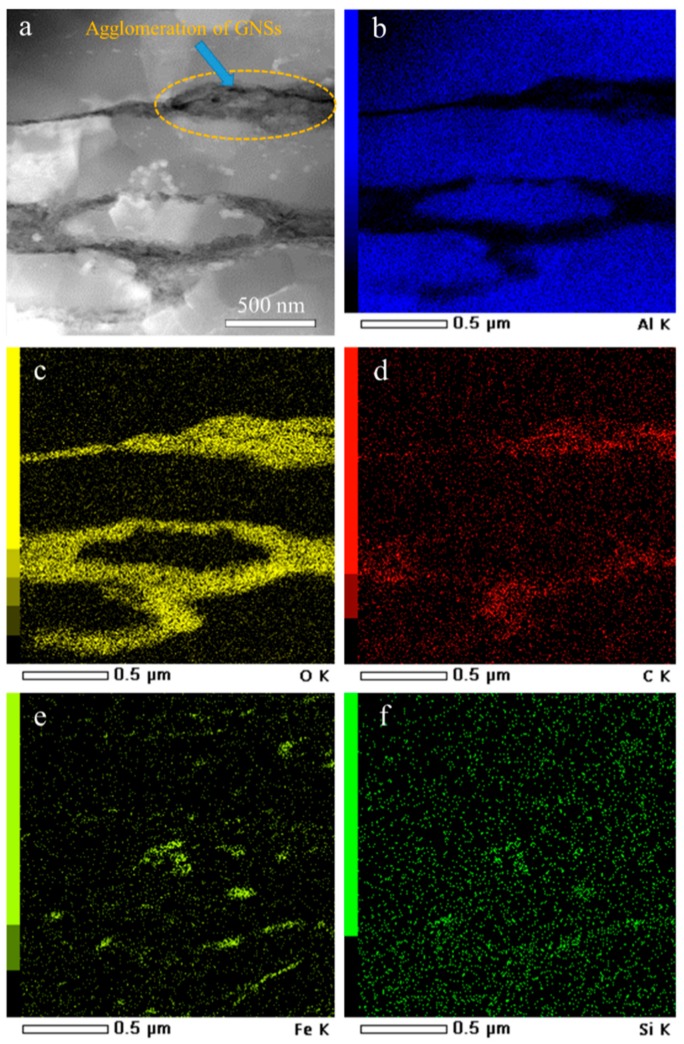
(**a**) Transmission electron microscopic image of Al-1 wt.% GNS and its element distribution map of (**b**) Al; (**c**) O; (**d**) C; (**e**) Fe; and (**f**) Si.

Local elemental maps of aluminum, oxygen, carbon, iron, and silicon in the 1 wt.% GNS sample are shown in Figure 7. These results are consistent with the SEM investigation and prove that along the former powder-particle boundaries, agglomeration of GNSs (in other words, formation of graphite by the attachment of GNSs) can be found with a black color; these could not be dispersed during ball-milling and ECAP. On the other hand, oxygen was probably related to the oxidization of few-layer graphene, which was investigated in the SEM results. In addition, it was proven that Fe and Si are located in small (Al-)Fe-Si particles as observed in both SEM and TEM. It should be noted that GNSs located in the aluminum matrix have significantly weaker intensities of the characteristic X-ray emissions due to their smaller volumes compared to those of GNS agglomerates. Therefore, EDS mapping cannot reveal the volume fraction of the GNSs distributed within the aluminum matrix. 

### 3.4. Density

In order to explore the consolidation level of Al-GNS nanocomposites, the density of samples was measured using the Archimedes method [58]. The results were compared to the theoretical density calculated according to the following equation [29]:

(1) $$\rho =\frac{100}{\frac{m_{m}}{\rho_{m}}+\frac{m_{r}}{\rho_{r}}}$$

where *ρ* is theoretical density, *m_m_* and *m_r_* are mass fraction of the matrix and reinforcement, respectively, and *ρ_m_* and *ρ_r_* are the density of matrix and reinforcement, respectively. It is worth mentioning that the densities for the Al powder and GNSs were 2.7 and 2.23 g·cm^−3^, respectively. The measured and theoretical densities of pure Al and the nanocomposites for the top and bottom parts of the consolidated billets are listed in Table 2. The results clearly show that an increase in GNS content results in a decrease in density. A similar effect of GNSs was previously observed in comparable material [59]. The difference between the theoretical and experimental density was negligible up to a GNS content of 0.5 wt.% and subsequently increased rapidly. A larger difference between densities (theoretical and experimental) in the 1 wt.% GNS sample might be related to the existence of micropores formed during processing [60]. The observed difference between the top and bottom parts of all samples could be explained by an effect of the back-pressure acting during ECAP. At the beginning of processing, the back-pressure resulting from friction between the billet and feed-out channel of the ECAP die was low. However, it continuously increased during the processing with the higher fractions of billet being processed. Consequently, hydrostatic pressure in the feed-in channel had to be increased in order to overcome increasing back-pressure, which had a positive effect on the consolidation process, and the density increased. It can be concluded that consolidation of the GNS nanocomposites was successful and almost full density was achieved for all samples except 1 wt.% of GNS. 

### 3.5. Mechanical Properties

#### 3.5.1. Hardness

**Figure 8:**
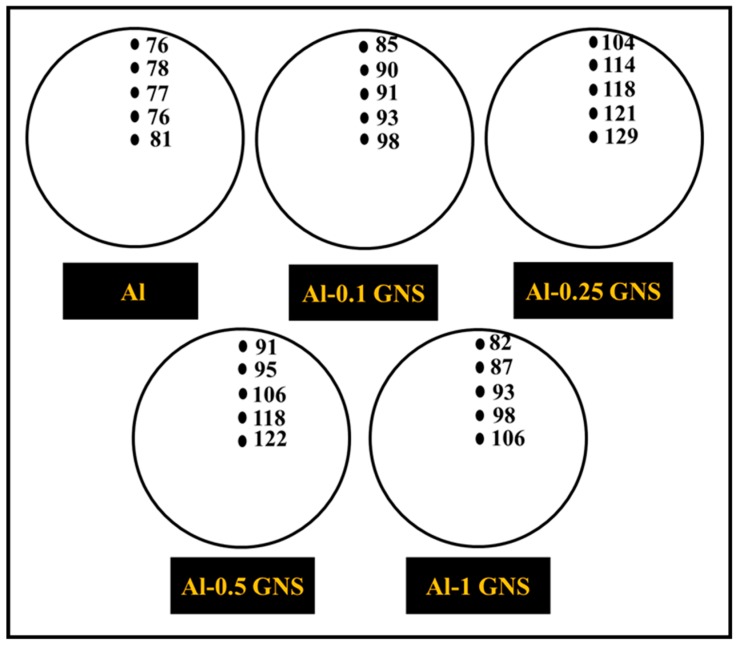
Variation of microhardness (HV) from the center to the edge of samples with different GNS content (Samples were prepared from the top part of the consolidated billet).

The results of microhardness measured in the top and bottom parts of all investigated samples are provided in Table 2. Increase in the GNS content had a clearly positive effect on the microhardness up to 0.25 wt.% GNSs (129 HV) followed by its rapid decrease. This increase could be attributed to the extraordinary mechanical properties of few-layer graphene, a stiff two-dimensional material, as a reinforcement of the matrix. The subsequent deterioration of microhardness with a further increase in GNS content was most probably caused by an increase in porosity, as shown in Table 2. In addition, SEM observation revealed that an increase in GNS content results in an increased volume fraction and thickness of the former particles’ boundaries, which are decorated by GNSs. Therefore, it is assumed that exceeding 0.25 wt.% of GNSs results in deterioration of mechanical properties due to an increase in porosity and a decrease of consolidation effectiveness due to the creation of GNS agglomerates along the particle boundaries. Furthermore, differences in microhardness between the top and bottom parts of the consolidated billet reflect differences between corresponding experimental densities. Figure 8 depicts the trend of hardness variation from the center to the edge of the specimen’s cross section. Hardness in the central part of all samples is the highest and decreases towards the periphery. This variation in hardness might be attributed to the variation of grain size, as shown in Figure 5. 

#### 3.5.2. Shear Stress

**Figure 9:**
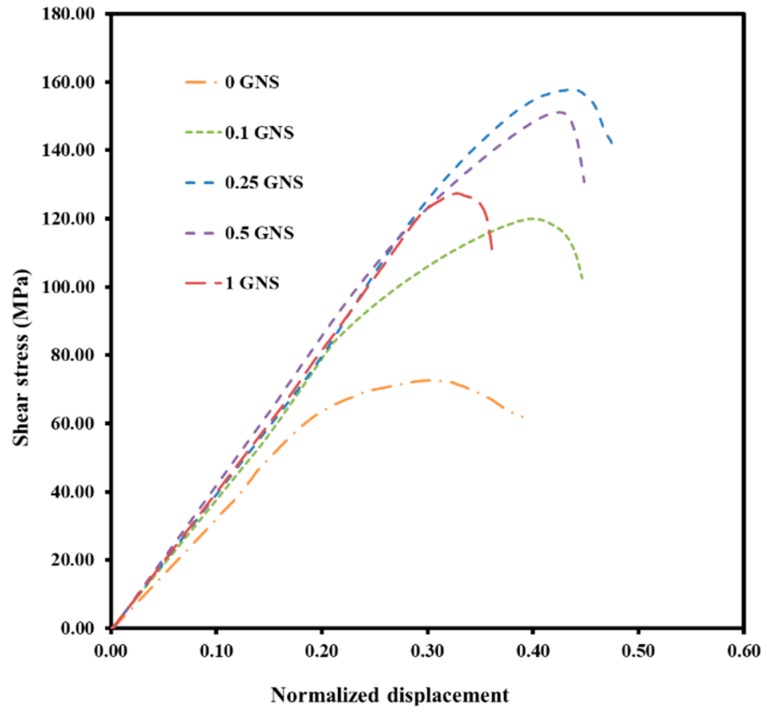
Engineering shear stress vs. normalized displacement of fabricated samples with different GNS weight percentages.

Figure 9 presents results from the shear punch tests of Al and Al-GNS nanocomposites. In addition, calculated values of shear yield stress (SYS), normalized displacement, and ultimate shear strength (USS) are provided in Table 3. It is clear that pure aluminum exhibited high elongation but low strength and hardening ability. The introduction of GNSs up to 0.25 wt.% into the aluminum matrix resulted in a significant increase in yield and ultimate shear strength. In addition, uniform normalized displacement was not deteriorated by the addition of 0.25 wt.% GNSs. However, further increases in GNSs to 0.5 wt.% and 1 wt.% resulted in deterioration of the above-mentioned parameters. 

Diffusion or chemical bonding, mechanical bonding, and Van der Waals bonding are the main interfacial bonding reported in metal-matrix composites [61]. Due to the low processing temperature (200 °C) used in the current investigation, the brittle Al_4_C_3_ phase did not form in the Al-GNS interface, and mechanical bonding resulting from surface wrinkles in the GNSs was the dominant interfacial bonding [62]. Therefore, better mechanical properties were expected. Moreover, ball-milling is not only able to distribute the GNSs ideally into the matrix, but also can improve the mechanical bonding and, consequently, stress transfer from the matrix to the reinforcements [63]. On the other hand, the bonding between graphene sheets was Van der Waals. When the GNS percentage was 1 wt.%, due to the creation of GNS agglomeration and more stacks of GNSs, Van der Waals interactions were much weaker than mechanical bonding and reduced the strength and uniform normal displacement. In contrast, in the sample with 0.25 wt.% GNS, because of thinner and exfoliated GNSs as well as good mechanical bonding with Al powders without any defects, the negative effect of the Van der Waals bonds was effectively lowered. Therefore, it is reasonable to assume that this sample possessed high strength and normalized displacement due to the easy transfer of stress. Also, the existence of microvoids in the 1 wt.% GNS composite is another reason for decreasing mechanical properties. In addition, TEM observation revealed significant refinement of the microstructure, which is a very effective strengthening mechanism according to the Hall–Petch relation [64]. 

Comparing the achieved results of Al-CNT samples prepared similarly by ECAP [27], Al-GNS composites exhibited higher shear strength. Shear strength of the Al-CNT material with an optimum composition was only 132 MPa after 8 passes through ECAP, while a shear stress of 158 MPa was attained in the Al-0.25 wt.% GNS with just one pass of ECAP. The reason for the better performance of Al-GNS samples most likely stems from a higher effectivity of GNSs for strengthening in comparison with other reinforcements such as CNT. GNSs are superior carbon reinforcements due to their planar shape, which provides good mechanical bonding and increases strengthening effectivity. In addition, during mechanical loading of the composite, a considerable portion of the load is transferred to the GNSs due to the extraordinary mechanical properties of few-layer graphene [45]. However, the effectivity of GNSs for strengthening significantly depends on the fabrication technique and, in particular, processing temperature. For a better comparison, the ultimate tensile strength (UTS) of ECAPed Al-GNS composites can be estimated using the Von Mises equation (σ = 1.73 τ) [65]. Comparing these values to the UTS of Al-GNS composites fabricated by hot-press [28] and hot-extrusion processes [26], the samples consolidated within this study are superior mainly due to the use of a lower processing temperature.

## 4. Conclusions 

In this study, Al-GNS nanocomposites with different weight percentages of GNSs were fabricated using a reliable, repetitive, ECAP process. The results confirmed that this method can be considered as a great and facile process to consolidate compacted powders in one pass with shear stress at 200 °C without the need of further sintering processes. Raman spectroscopy of the sample with 0.25 wt.% GNSs demonstrated that the GNSs were exfoliated with reasonable damage or disordering. All samples except the 1 wt.% GNS composites were fully dense (>98%) and maximum hardness, ultimate shear strength, and uniform normal displacement was attributed to the 0.25 wt.% GNS composite. According to microstructural characterization by TEM and SEM, it is likely the ultra-grain refining of the matrix and the presence of exfoliated few-layer graphene as a great carbon reinforcement that created good mechanical bonding during the ball-milling and ECAP processes. However, a decrease in the mechanical properties of the 1 wt.% GNS sample might be attributed to microporosities in the matrix and agglomerated GNSs, which were also created along the former powder-particles boundaries.

## Figures and Tables

**Table 1:** Summary of information related to the Raman spectroscopy.

-	Pure GNSs	Al-0.25 GNSs	Al-0.25 GNSs Bulk	Al-0.5 GNSs	Al-1 GNSs
RSGP	1571.235	1590.780	1591.899	1590.780	1590.780
ID/IG	0.3033	0.6765	0.864	0.722	0.960

RSGP: Raman shift of G peak (cm^−1^). ID/IG; intensity ratio of D (ID) and G (IG) peaks.

**Table 2:** Theoretical/experimental densities and the microhardness (Vickers) of pure Al and its nanocomposites for the top and bottom of each sample. The measurement was performed in the center of the billets’ cross-section.

Sample	Hardness (HV)		Density (Top)			Density (Bottom)		
-	Top	Bottom	Theo.	Exp.	Per. (%)	Theo.	Exp.	Per. (%)
Pure Al	81 ± 2	79 ± 1	2.700	2.69 ± 0.07	99.62	2.700	2.69 ± 0.04	99.62
Al-0.1 wt.% GNSs	98 ± 1	89 ± 2	2.699	2.69 ± 0.05	99.66	2.699	2.67 ± 0.08	98.92
Al-0.25 wt.% GNSs	129 ± 5	120 ± 3	2.698	2.68 ± 0.09	99.33	2.698	2.65 ± 0.05	98.22
Al-0.5 wt.% GNSs	122 ± 2	116 ± 3	2.697	2.67 ± 0.03	98.99	2.697	2.62 ± 0.02	97.14
Al-1 wt.% GNSs	106 ± 3	95 ± 4	2.694	2.58 ± 0.06	95.75	2.694	2.55 ± 0.04	94.65

Theo.: Theoretical; Exp.: Experimental; Per.: Percentage.

**Table 3:** Information related to shear yield strength (MPa), ultimate shear strength (MPa), uniform normal displacement (mm/mm), and uniform plastic normal displacement (mm/mm) of fabricated samples with different GNS weight percentages.

Sample	Shear Yield Strength (MPa)	Ultimate Shear Strength (MPa)	Uniform Normal Displacement (mm/mm)	Uniform Plastic Normal Displacement (mm/mm)
Pure Al	64 ± 2	73 ± 3	0.32 ± 0.02	0.16 ± 0.01
Al-0.1 wt.% GNSs	85 ± 1	119 ± 1	0.40 ± 0.03	0.17 ± 0.01
Al-0.25 wt.% GNSs	124 ± 3	158 ± 2	0.44 ± 0.01	0.14 ± 0.03
Al-0.5 wt.% GNSs	105 ± 4	151 ± 3	0.42 ± 0.02	0.13 ± 0.02
Al-1 wt.% GNSs	98 ± 5	127 ± 4	0.32 ± 0.04	0.10 ± 0.03
